# Combined Spatio-Temporal Impacts of Climate and Longline Fisheries on the Survival of a Trans-Equatorial Marine Migrant

**DOI:** 10.1371/journal.pone.0040822

**Published:** 2012-07-16

**Authors:** Raül Ramos, José Pedro Granadeiro, Marie Nevoux, Jean-Louis Mougin, Maria Peixe Dias, Paulo Catry

**Affiliations:** 1 Eco-Ethology Research Unit, Instituto Superior de Psicologia Aplicada, Lisboa, Portugal; 2 Centre for Environmental and Marine Studies, Museu Nacional de História Natural e da Ciência, Universidade de Lisboa, Lisboa, Portugal; 3 Mammal Research Institute, Department of Zoology and Entomology, University of Pretoria, Hatfield, South Africa; 4 Laboratoire de Zoologie, Muséum National d’Histoire Naturelle, Paris, France; Hawaii Pacific University, United States of America

## Abstract

Predicting the impact of human activities and their derivable consequences, such as global warming or direct wildlife mortality, is increasingly relevant in our changing world. Due to their particular life history traits, long-lived migrants are amongst the most endangered and sensitive group of animals to these harming effects. Our ability to identify and quantify such anthropogenic threats in both breeding and wintering grounds is, therefore, of key importance in the field of conservation biology. Using long-term capture-recapture data (34 years, 4557 individuals) and year-round tracking data (4 years, 100 individuals) of a trans-equatorial migrant, the Cory’s shearwater (*Calonectris diomedea*), we investigated the impact of longline fisheries and climatic variables in both breeding and wintering areas on the most important demographic trait of this seabird, i.e. adult survival. Annual adult survival probability was estimated at 0.914±0.022 on average, declining throughout 1978–1999 but recovering during the last decade (2005–2011). Our results suggest that both the incidental bycatch associated with longline fisheries and high sea surface temperatures (indirectly linked to food availability; SST) increased mortality rates during the long breeding season (March-October). Shearwater survival was also negatively affected during the short non-breeding season (December-February) by positive episodes of the Southern Oscillation Index (SOI). Indirect negative effects of climate at both breeding (SST) and wintering grounds (SOI) had a greater impact on survival than longliner activity, and indeed these climatic factors are those which are expected to present more unfavourable trends in the future. Our work underlines the importance of considering both breeding and wintering habitats as well as precise schedules/phenology when assessing the global role of the local impacts on the dynamics of migratory species.

## Introduction

Understanding population dynamics of long-lived marine vertebrates is essential to identify relevant human impacts for the sustainability of our oceans [1], [2]. Due to the complexity of their annual cycles, this knowledge is particularly important for long-distance migrants as these anthropogenic threats might impact them over a larger geographical range, while breeding, migrating or at their wintering grounds [3], [4].

We have now clear evidences that human activities and resulting global changes are strongly impacting marine ecosystems [2], [5], [6]. A major threat for marine top predators is the increasing industrial fisheries occurring in most of our oceans [7], [8]. Effects of these fisheries on apical species may be negative, increasing incidental mortality [9]–[11], impoverishing food webs and reducing fish stocks [12], but also positive either directly through discards which provide additional food [13], [14] or indirectly by removing larger predatory fish (i.e. competitors). From the conservation point of view, fishery sustainability represents a sensitive, socio-economic issue and data regarding these collateral effects on marine top predators is scarce and poorly reported by the competent authorities [15]–[17], generating a poor knowledge on the real impact of commercial fisheries on marine megafauna. Global warming is also inducing changes in the distribution and abundance of marine prey and will therefore affect the dynamics of their predators [18]–[21]. Several long-term studies have documented links between climate and population dynamics through both local fluctuations in oceanographic parameters (e.g. sea surface temperature, SST; [22]) and large-scale cyclic patterns (e.g. Southern Oscillation Index, SOI; [23], [24]). The fact that the increasing global warming especially impacts polar environments [25], [26], might bias the bulk of climate-induced demography research towards Arctic and Antarctic species. In this sense, little is known about the potential effects of local and large-scale climatic phenomena on the productivity of temperate to tropical oceanic water masses.

Assessing the precise interactions between these changes and marine predator dynamics will therefore be critical for effective conservation management. In this sense, long-lived predator species are rather the most endangered and sensitive group of animals to environmental perturbation due to their extreme life history traits (e.g. high survival, low fecundity and an usually considerable degree of specialization; [9], [27]). Moreover, migratory predators, inhabiting very different water masses throughout their annual cycles, are particularly challenging in this respect (e.g. [28]). For instance, environmental conditions could have a strong impact on these migratory species not only at the breeding grounds but also along their migration routes or at their wintering grounds, when individuals from a variety of breeding origins congregate into common migratory corridors and wintering areas. Hence, the ability to identify and quantify the respective roles of climate and human activities in both breeding and wintering grounds of these long-lived top predators is important in the field of marine biological conservation.

The sophistication of statistical modelling techniques and the increasing availability of environmental data makes possible to integrate both climate and human effects on powerful demographic models to ultimately build realistic scenarios of the impact of future environmental changes on populations of marine organisms (e.g. [29], [30]). Spatio-temporal impacts of harmful processes on population dynamics have been studied in large marine species (e.g. albatrosses and marine mammals), but this has seldom been done in smaller migratory predators despite the fact they might be highly relevant in explaining energy flow and food web structuring in marine environments [31]–[33]. In addition, precise movements and foraging locations for these highly mobile predators are often unknown, notably in winter when many of these species remain unavailable to most researchers very far from their breeding colonies. In this sense, the Cory’s shearwater (*Calonectris diomedea*) represents a good model species as (1) it is a medium-sized long-lived marine top predator, (2) it breeds colonially at temperate to sub-tropical latitudes, (3) it carries out an annual trans-equatorial migration, (4) its survival is known to be affected by climate [34]–[37], and (5) several of its populations may be threatened by longline fisheries [38]. We took advantage of a capture-recapture monitoring carried out on the world’s largest colony of Cory’s shearwater over 34 years (from 1978 to 2011, with an in-between four-years gap), (a) to model its adult survival and (b) to assess the potential effects of fishery activity and (c) climate fluctuations on this long-lived migratory predator. More novelty, in order to select the most appropriate environmental variables that should be tested as candidate predictors of survival, detailed information on spatial and temporal distribution of the study population was obtained along four years by tracking 100 of their long-distance migrations.

## Methods

### Study Site and Data Collection

**Figure 1:**
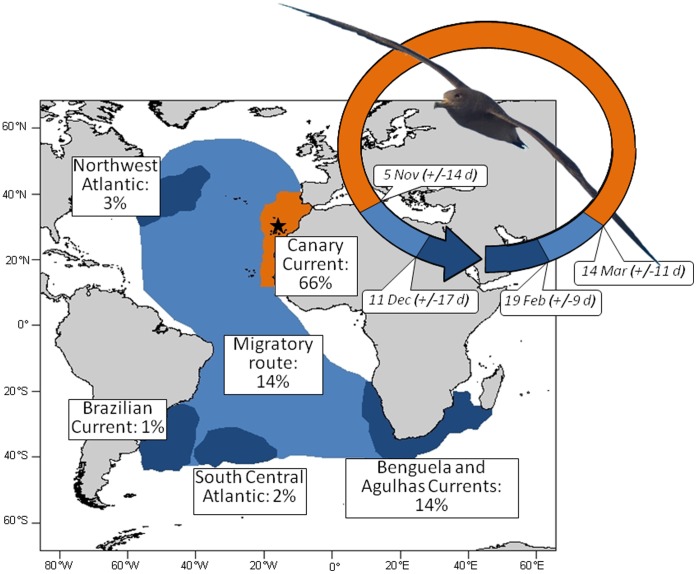
Distribution of Cory’s shearwaters from Selvagem Grande Island (star) throughout the annual cycle. Schematic annual phenology (starting 1st January) and annual distribution of 100 Cory’s shearwaters tracked with geolocators between 2006 and 2009. Coloured areas encompass bird positions during the breeding and wintering seasons (in orange and dark blue, respectively) and the overall distribution during the migration periods (i.e., when commuting between breeding and wintering areas; in light blue). The main wintering areas were associated with Benguela and Agulhas Currents (n = 72 individuals), central South Atlantic (n = 11), Brazil-Malvinas confluence region (n = 8), northwest Atlantic (n = 4) and Canary Current (n = 5). Estimated proportion of time spent in each area by the whole adult population is shown in white panels. Note that the Canary Current includes all breeding positions as well as the few wintering ones. Photo credit R. Ramos.

**Figure 2:**
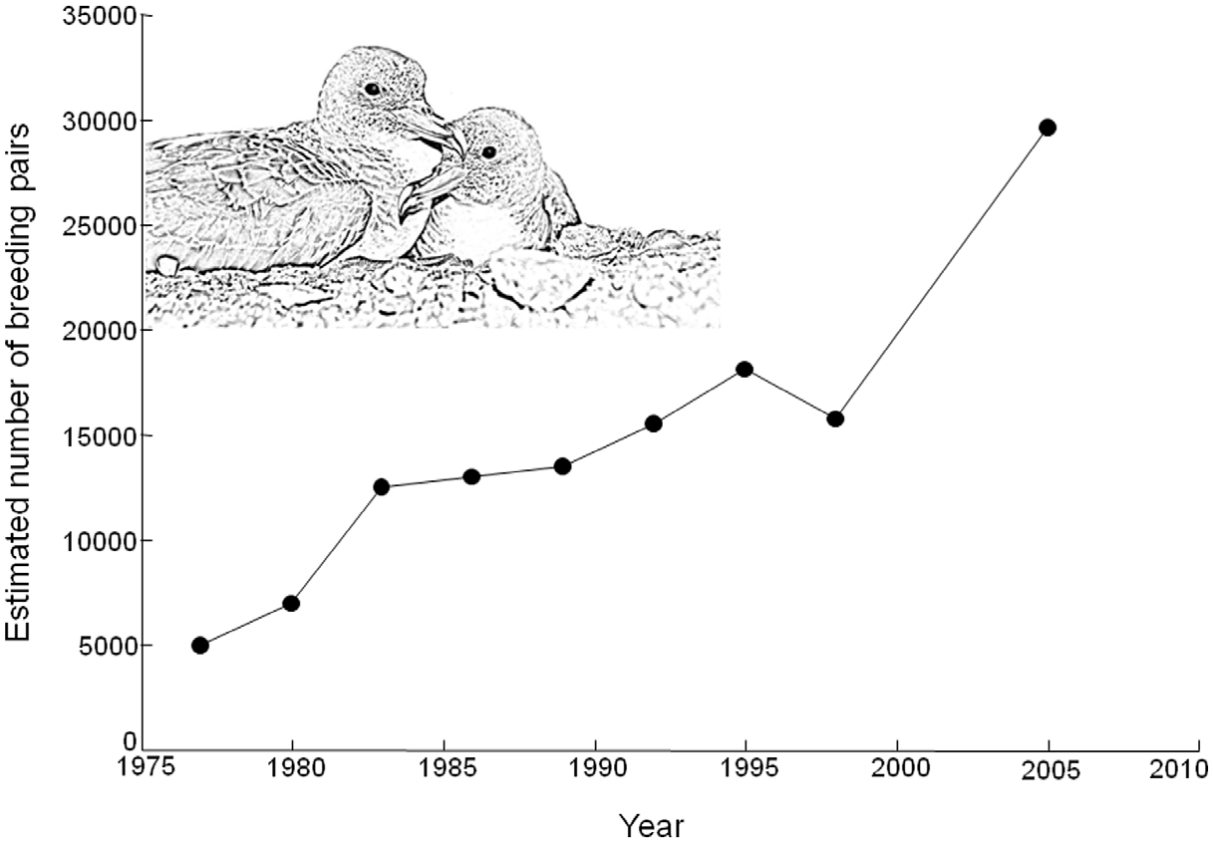
Estimated breeding population of Cory’s shearwaters at Selvagem Grande Island along the sampled period. Data from Mougin et al. 2000 and Granadeiro et al. 2006. Photo credit R. Ramos.

The island of Selvagem Grande (30°09′N, 15°52′W; Fig. 1) holds the largest known breeding colony of Cory’s shearwater in the world [39]. There are some historical records of its exploitation at this remote location, with Cory’s shearwater eggs and chicks being intensively exploited for food, oil and feathers through the first two thirds of the 20^th^ century [40]. Constant harvests of chicks but also a few severe killings of adults in the 1970’s, reduced this population from *ca.* 100,000 breeding pairs (crude estimations at the beginning of the 20^th^ century) to only 5,000 pairs in 1977 [41]. Since then, and due to the establishment of the Selvagens Islands Nature Reserve and a permanent vigilance, the shearwater population has increased and was estimated at *ca.* 29,540 breeding pairs in 2005 (Fig. 2; [39]). During the breeding season (March-October), Cory’s shearwaters from Selvagem Grande forage to a large extent in the Canary Current, along the Moroccan and Western Sahara coast where productivity is high, owing to the enrichment of the surface waters by a strong upwelling [42], [43]. Most of these trans-equatorial migratory shearwaters congregate in the South Atlantic Ocean during the non-breeding season (November-February), the Benguela Current being one of the most used wintering sites [44], [45].

Each year since the 1978 breeding season, new individuals found nesting at study sites within the colony and all their chicks were ringed with a monel band. The presence/absence of each ringed individual in the colony was recorded annually from 1978 by consecutive burrow visits during early incubation (early June). The analysed dataset is composed by (1) a continuous period of 22 years monitoring data (from 1978 to 1999) from a sample of *ca.* 500 individually numbered nests (3,227 individuals), and, (2) a period of eight years (from 2004 to 2011) coming from a different sample of 358 numbered nests (1,330 individuals), with no data collected in-between.

### Tracking Data

Additionally, to choose the most appropriate and relevant environmental predictors for Cory’s shearwater survival, we first characterized the phenology and annual distribution of birds breeding at Selvagem Grande Island. We tracked their trans-equatorial migrations by deploying leg-mounted 3.6-g geolocators (mk7 model, developed by British Antarctic Survey, [46]) at the end of breeding seasons 2006, 2007, 2008 and 2009 (August/September). In the beginning of the following breeding seasons (April/June), we recovered complete data from 100 geolocators. Geolocators provide two positions per day based on light levels (one at local midday and other at local midnight), with an accuracy of approximately 186±114 km [47]. Data were analysed using TransEdit (to check for integrity of light curves and to fit dawn and dusk times; [46]) and Birdtrack software (to estimate the latitude from day length and longitude from the time of local midday relative to Greenwich Mean; [46]). We assumed a sun elevation angle of −4.5 degrees, based on known positions obtained during ground-truthing of loggers carried out before and after deployment. Unrealistic positions (those resulting from interference of light curves at dawn or dusk, or around equinox periods) were removed from the analyses.

### Environmental and Fishery Data

We chose a series of climatic and fishery-related indices with biological interest for Cory’s shearwaters to explore their relationship with adult survival (Table 1). We extracted monthly values of all indices specifically for the Canary Current (20°–35°N, 10°–20°W) to evaluate their effect on the breeding ground, and for the Benguela (15°–40°S, 5°–25°E) and Agulhas (15°–40°S, 25°–45°E) Currents to assess their impact on the non-breeding grounds as described by tracking data (Fig. 1 and Appendix S1). In order to integrate the breeding and non-breeding periods separately, we averaged monthly data over April-September and December-February, respectively.

**Table 1:** Questions addressed concerning the impact of fisheries and climate on Cory’s shearwater survival.

Question of interest	Covariate response in the breeding ground	Covariate response in wintering grounds	Potential effect on survival
Are longline fisheries having an impact on survival?	LL_CC_	LL_BA_	Direct, negative
Is SST affecting shearwater survival?	SST_CC_	SST_BA_	Indirect, positive or negative
Does SOI have an impact on its survival?	–	SOI	Direct or indirect, negative

LL longlining effort; SST Sea Surface Temperature; SOI Southern Oscillation Index; subindex _CC_ Canary Current; subindex _BA_: Benguela and Agulhas Currents.

Note that specific covariate responses (longlining activity: LL, sea surface temperature: SST, and Southern Oscillation Index: SOI) in breeding and wintering grounds were tested. Potential effects of these covariates on survival were also predicted as direct when the covariate in itself influences survival, indirect when it is though a trophic cascade effect for instance, positive when the effect of the covariate increases survival probability, and negative when that effect damages survivorship.

In most cases, fishery-related indices are expected to affect directly adult survival, with longlining affecting negatively seabirds through incidental bycatch (e.g. [48], [49]). In particular, foraging areas exploited by Cory’s shearwater both in breeding and wintering periods are thought to largely overlap with areas frequented by longlining fishery vessels [38], [50], [51]. Thus, we tested the effect of longlining effort (LL) by extracting the number of hooks used by tuna longliners in specific areas and seasons (Appendix S1). Longlining data was obtained from International Commission for the Conservation of Atlantic Tunas (ICCAT) website (http://www.iccat.es/en/accesingdb.htm), which offers small scale (5°×5° squares) monthly catch and fishing effort indices.

Climatic fluctuations are also suspected to affect seabird mortality either directly through storminess (e.g. [34]), or after a temporal lag through an indirect mechanism (e.g. [52]), where climate first affects primary production, and then integrates along the trophic web up to top predators [53]. Firstly, we explored potential effects of the Southern Oscillation Index (SOI, available at http://www.esrl.noaa.gov/psd/data/correlation/soi.data), which reflects El Niño/La Niña large-scale oscillations through changes in sea level pressure between the south-eastern and south-western Pacific waters. Although the effect of the SOI is most pronounced in the south-eastern Pacific Ocean, other southern marine ecosystems such as the Southern Atlantic where most Cory’s shearwaters winter seem also affected by sustained positive SOI values (i.e. La Niña episodes; [54]). Secondly, we tested a local index, the Sea Surface Temperature (SST, available at http://badc.nerc.ac.uk/view/badc.nerc.ac.uk_ATOM_dataent_hadisst, at 1° spatial resolution, HadISST 1.1; Hadley Centre, British Atmospheric Data Centre), which provides information on oceanographic conditions on a finer geographical scale and which may ultimately structure the trophic web. Finally, we considered both SOI and SST variables lagged one (breeding to non-breeding, or vice versa), two (i.e. one entire year), three or even four seasons (see Appendix S1 for details). By these means, we investigated the potential long-lasting effects of these environmental variables through the whole trophic web on survival.

To reduce the number of explanatory variable and avoid spurious relationships (errors of type I, [55]), we either combined correlated variables of the same nature (i.e. units) by averaging all respective months (e.g. SST in the breeding ground for four consecutive seasons and SOI for the non-breeding and the previous breeding seasons) or we performed a Principal Component Analyses (PCA) when the correlated variables were of a different nature (e.g. LL with all SST in the non-breeding grounds). We retained the first and second PCs for that last synthetic covariate (denoted by PC1LLSST and PC2LLSST, respectively) which accounted for 47.7% and 28.8% of the variation of the four original covariates, respectively. This reduced set of six variables is detailed in Appendix S1.

### Demographic Modelling

Demographic parameters were estimated with capture-mark-recapture (CMR; [56]) models, using M-Surge version 1.8 [57] and a total of 4,555 adult capture-recapture histories, over the 1978–2011 period. We started with the Cormarck-Jolly-Seber (CJS) model where survival (Φ, probability that a shearwater alive at year *t* survives at year *t*+1) and capture (p, probability that a shearwater alive and present at the breeding colony at year *t* is caught during the year *t*) were time (*t*) and group (*sex*) dependent. The fit of the general model to the data was investigated with goodness-of-fit (GOF) tests for each period (1978–99 and 2004–11) and sex using program U-Care version 2.2 [58]. Model selection was done using the Akaike Information Criterion corrected for small sample size and overdispersion (QAICc; [59]). When comparing two models, if ΔQAICc >2, the preferred model is the one with the smallest QAICc value (i.e. the most parsimonious model in terms of the number of parameters and model deviance; [56]). Along the modelling, capture probability was fixed to zero for the period 2000–2003 in all the individuals, and for the period 2004–2011 in those individuals sampled during the first period (i.e. 1978–1999) to account for changes in the monitoring protocol over the course of the study. To test and evaluate the impact of (uncorrelated) climate and fishery covariates on adult survival, we progressively built models from the best time dependent model including one covariate at a time. We started testing the potential linear effect of longline fisheries (direct mortality) on survival and we then tested for linear and non-linear (quadratic) effects of the climate on seabird demography. We also tested whether the impact of each of these covariates was constant over the entire period 1978–2011 (one slope) or whether it differed between 1978–1999 and 2004–2011 periods (two slopes). By doing so, we aimed to disentangle whether these variables had changed their potential effect between the two considered periods. The ability of each covariate to describe significant variation in survival was assessed using analysis of deviance tests (ANODEV: *F*-test statistic with *n*_cov_ and *n*-*n*_cov_-1degrees of freedom, where *n*_cov_ represented the number of covariates included and *n* was the number of parameters of the time dependent model; [60]) and the effects of these covariates were quantified using an approximated *R^2^*statistic [61]: *R^2^* = DEV(*M_cst_*)-DEV(*M_cov_*)/[DEV(*M_cst_*)-DEV(*M_t_*)], where DEV(*M_cst_*), DEV(*M_cov_*) and DEV(*M_t_*) are the respective deviances for the models constant, with covariate(s) and time dependent. While modelling the effect of the covariates, *M_cst_* were selected according to the aim of the test (see Table 3 for details).

## Results

### Annual Phenology and Distribution Gathered from Geolocator Data

Movements of tracked Cory’s shearwaters were easily classified into frequent foraging trips around the breeding colony (including the Canary Current), rapid, long-distance migratory movements, and persistent presence in a well defined non-breeding ground, by combining location data and date. Most birds wintered in five broad areas (Fig. 1), associated with the Benguela and Agulhas Currents (72% of the individuals), central South Atlantic (11%), the Brazilian Current (8%), northwest Atlantic (4%) and the Canary Current (5%). On average, they left the colony the first fortnight of November (mean departure date: 5 November+/−14 days), and took 36 days to reach their major destination (mean arrival date: 11 December+/−17 days). Birds left their non-breeding areas around mid-February (19 February+/−9 days) and arrived at Selvagem Grande three weeks later (14 March+/−11 days). Overall, Cory’s shearwaters from Selvagem Island spend 66.3% of their annual time in the breeding grounds, only 13.8% of the time migrating and 19.9% in one of their non-breeding areas, mainly in the South Atlantic Ocean (Fig. 1). Later, these dates, time expenditure and precise locations along the year allowed us defining precise spatio-temporal delimitations of several environmental indices which were likely to affect Cory’s shearwater survival.

### Goodness of Fit Test

The GOF test indicated a severe lack of fit of the umbrella model (χ^2^_316_ = 1783.7, *P*<0.0001), coming from the presence of both transient and trap dependence effects [62]. The first effect suggested the presence of transients, i.e. prospecting or inexperienced animals from other locations, in both periods and sexes (test 3.SR in Table 2). Transience violates the CJS model assumption of equal survival between newly and previously marked individuals. In the case of high capture probabilities, transient individuals can be efficiently deleted by suppressing the first recapture of all animals [63], [64]. After doing so, test 3.SR was not significant anymore for any of the periods or sexes (Table 2). The positive trap-dependent effect (test 2.CT in Table 2), which indicates that individuals captured on one occasion were more likely to be captured on the following occasion than others was accounted by splitting capture-recapture histories (using U-Care software) and implementing a trap-dependence model (where capture probability results from a Markovian dependence on previous capture, denoted by *m*; [65]). Finally, our modelling based on Φ_sex*t_ p_m*sex*t_ was also corrected with a variance inflation factor *c-hat* to take into account the remaining lack of fit (χ^2^_203_ = 354.7, *P*<0.0001), calculated as the χ^2^ statistic over its number of degrees of freedom (*c-hat* = 1.746; [56]).

**Table 2:** Results of goodness-of-fit (GOF) tests of CJS model (Φ*_t_* p*_t_*), for each period (1978–99 and 2004–11) and sex. individual was removed to account for transience.

		Test 3SR			Test 3SM			Test 2CT			Test 2CL						Sum of Tests				
		df	χ^2^	*P*	df	χ^2^	*P*	df	χ^2^	*P*	df		χ^2^		*P*		df		χ^2^		*P*
Complete data set																					
1978–1999	males	20	36.3	0.014	42	138.3	0.000	19	360.5	0.000	52		101.5		0.000		133		636.7		0.000
	females	20	71.1	0.000	39	113.3	0.000	19	516.2	0.000	54		101.0		0.000		132		801.6		0.000
2004–2011	males	6	41.1	0.000	8	36.3	0.000	5	61.2	0.000	6		33.6		0.000		25		172.1		0.000
	females	6	18.7	0.005	8	38.9	0.000	5	102.4	0.000	7		13.3		0.066		26		173.3		0.000
After removing first encounter																					
1979–1999	males	19	15.8	0.674	32	53.2	0.011	18	259.8	0.000	35	64.7		0.002		104		393.4		0.000	
	females	19	20.7	0.352	33	83.1	0.000	18	378.7	0.000	37	73.2		0.000		107		555.7		0.000	
2005–2011	males	5	9.0	0.110	5	8.3	0.143	4	35.0	0.000	3	8.7		0.034		17		60.9		0.001	
	females	5	3.4	0.646	6	5.5	0.482	4	60.5	0.000	4	9.1		0.058		19		78.4		0.000	

Two different datasets are used: a complete dataset with all the encounters and a reduced dataset where the first recapture of every.

Tests 3 (3SR and 3SM) check the homogeneity of recapture histories while tests 2 (2CT and 2CL) examine the independence between last release and next recapture (Burnham & Anderson 1998); df degrees of freedom; χ^2^ Pearson’s chi-squared statistic; *P* significance of the χ^2^ test.

### Estimating Demographic Parameters through the 1978–2011 Period

**Figure 3:**
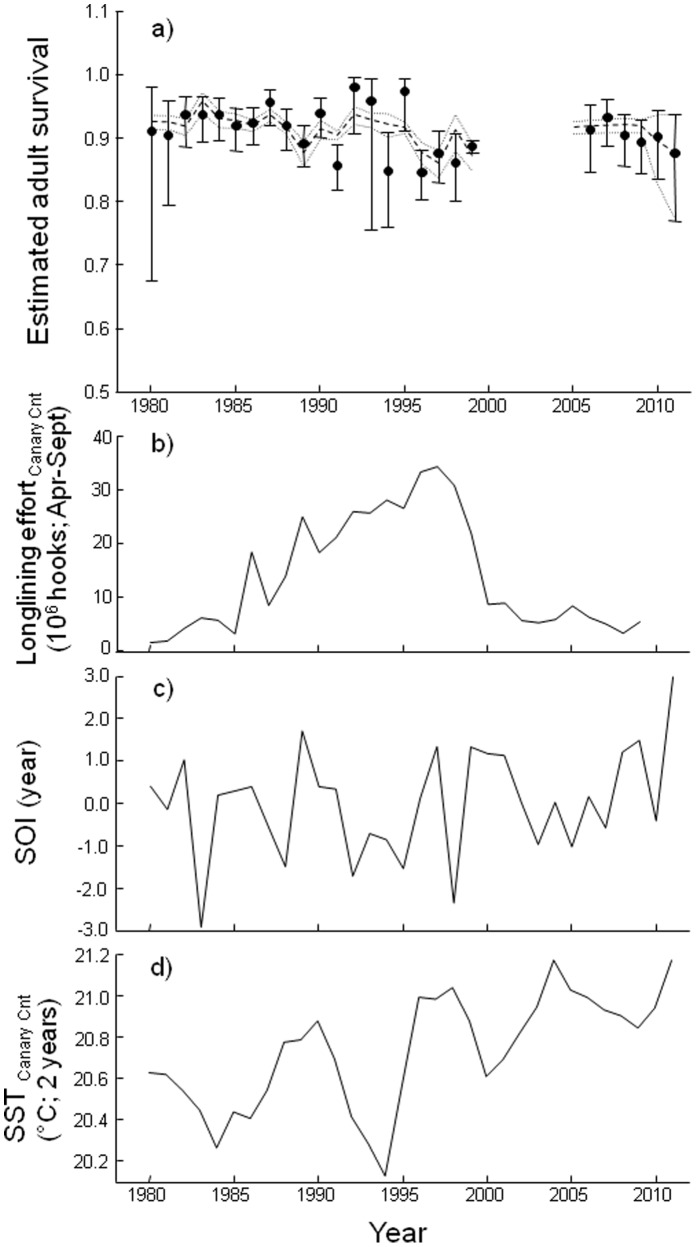
Annual variation of adult survival probabilities and the selected covariates for the period 1980–2011. Variation in survival rates of adults (a) and the selected covariates: (b) longlining effort in the Canary Current during the breeding, (c) annual SOI and (d) SST in the Canary Current (2 years averaged) are shown separately. Survival estimates come from the time-dependent model (Φ*_t_* p*_m+t_*; in black dots and CI in solid bars) and from the selected model with the covariates (Φ*_LLccbr+SOIyr+SSTcc2yr_* p*_m+t_*; in dense dashed line and CI in light dashed lines).

**Figure 4:**
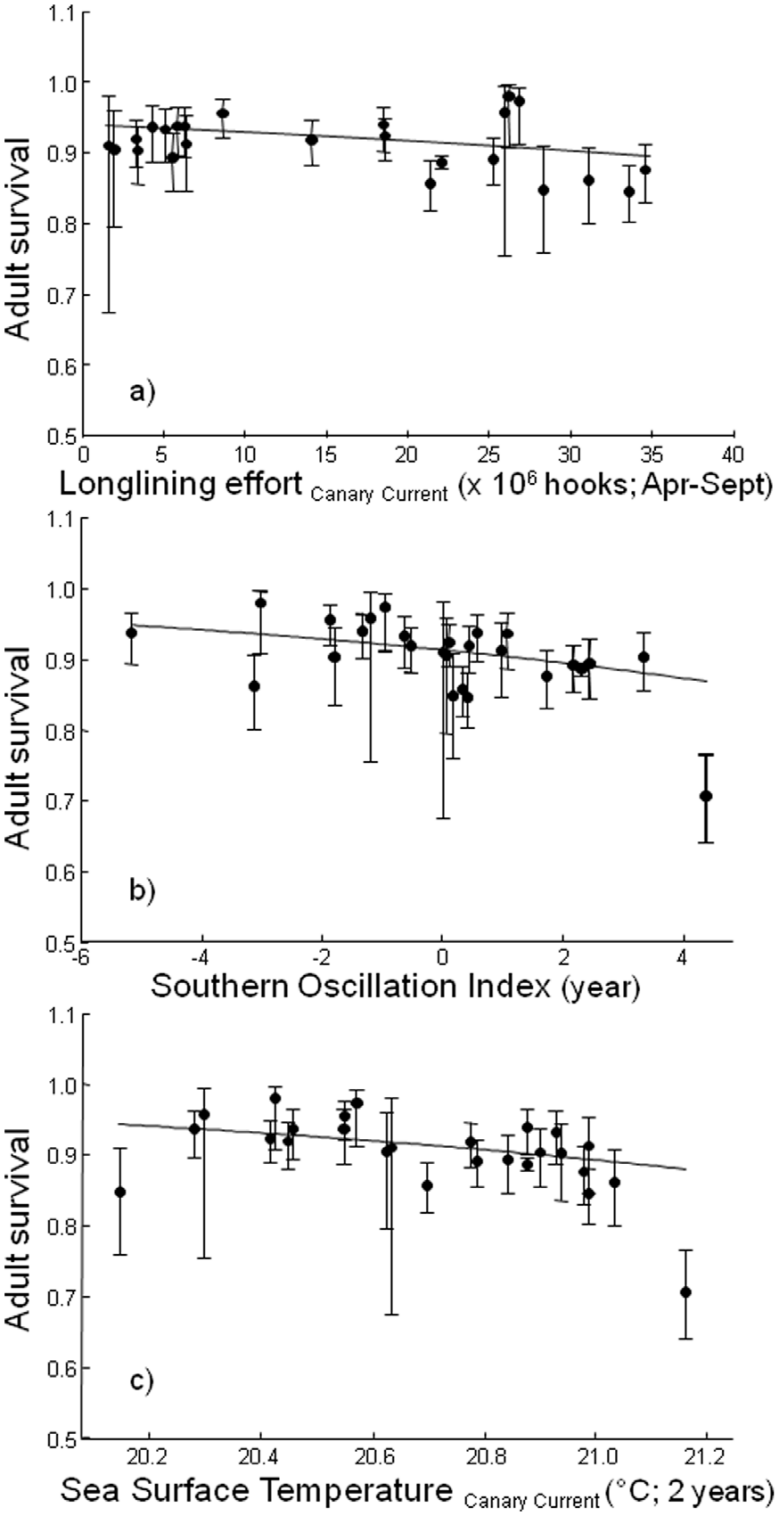
Relationship between annual adult survival probabilities and the selected covariates from the best CMR model. (a) Longlining effort in the Canary Current during the breeding, (b) annual SOI and (c) SST in the Canary Current (2 years averaged) are depicted against survival estimates (mean in black dots and asymmetric CI in solid bars estimated from the model Φ*_t_* p*_m+t_*). Regression lines estimated from model 24 in Table 3.

We started testing in the umbrella model (Φ_sex*t_ p_m*sex*t_) whether survival and capture probabilities varied with sex and/or time (Table 3). The lowest QAICc was obtained for a model with an additive effect of time on trap-dependence categories (additive trap effect) for capture probability and time-dependent survival (model 7; Fig. 3a). Survival probability was estimated as Φ = 0.915 (SE = 0.037, range = 0.909–0.921) from the constant model (model 8). We then assessed the effect of covariates using the model structure of that selected time-dependent model as a starting point (model 7; Table 3). Searching for direct causes of mortality (models 9–14), we detected a significant negative effect of the longlining effort on the Canary Current during the breeding period on survival (LL_CC_br, model 9, ANODEV = 5.772, *P_ANODEV_* = 0.024), explaining 20.8% of the variation. We then tested for potential additive effects of climatic parameters on Cory’s shearwater survival (models 15–26): in model 15, we noted a significant negative impact of SST in the Canary Current on survival estimates (SST_CC_2yr, ANODEV = 6.623, *P_ANODEV_* = 0.005) although in model 18, a more significant negative effect of annual SOI was found impacting on survival parameters (SOIyr, ANODEV = 7.937, *P_ANODEV_* = 0.002, *R^2^* = 0.425). In model 24, additively to LL_CC_br and SOIyr, the integrative covariate of SST of the Canary Current was still affecting seabird demography (SST_CC_2yr, ANODEV = 3.984, *P_ANODEV_* = 0.021, *R^2^* = 0.498; Fig. 3). The relevance of both climatic variables was more apparent when they were considered in a linear trend, suggesting therefore weak non-linear effects of climate on Cory’s shearwater survival (models 16, 19 and 25). In addition, none of the models including different slopes for 1980–1999 and 2005–2011 periods were preferred when tested against the model with a single slope (although models 10 and 20 were close to), suggesting similar effects of the covariates along the two periods. Finally, none of the confidence intervals of the selected covariates included zero, suggesting that all three covariates, i.e. LL_CC_br, SOIyr and SST_CC_2yr contributed negatively and significantly (−0.104±0.040, −0.212±0.081, −0.137±0.058, respectively) to year-to-year variations in survival throughout the period 1980–2011 (Fig. 4).

**Table 3:** Modelling capture (p) and survival (φ) probabilities in the Cory’s shearwater breeding at Selvagem Grande Island and the effects of covariates on survival.

n°	Model	np	DEV	QAICc	ΔQAICc	P_ANODEV_	M_cst_
Modelling capture probability (p)							
1	φ (sex*t) p (m*sex*t)	148	28296.6	16502.5	120.3		
2	φ (sex*t) p (m*t)	100	28353.1	16438.9	56.7		
3	φ (sex*t) p (*m*)	56	29609.1	17070.3	688.0		
4	φ (sex*t) p (*m1(·) m2(t)*)	77	28601.5	16535.2	153.0		
5	φ (sex*t) p (*m1(t) m2(·)*)	74	29437.2	17007.8	625.6		
**6**	**φ (** sex*t **) p (** ***m+t*** **)**	**79**	**28383.6**	**16414.4**	**32.1**		
Modelling survival probability (φ)							
**7**	**φ (** ***t*** **) p (** ***m+t*** **)**	**53**	**28418.3**	**16382.2**	**0.0**		
8	φ (·) p (*m+t*)	29	28553.4	16411.6	29.4		
Modelling covariates in survival							
Direct mortality							
**9**	**φ (** ***LL_CC_br _1980–2011_*** **) p (** ***m+t*** **)**	**31**	**28525.5**	**16399.6**		**0.024**	**8**
10	φ (*LL_CC_br _1980–99+2005–11_*) p (*m+t*)	32	28512.0	16393.9		0.068	9
11	φ (PC1*LLSST_BA1980–2011_*) p (*m+t*)	31	28553.1	16415.5		0.795	8
12	φ (PC1*LLSST_BA1980–99+2005–11_*) p (*m+t*)	32	28552.8	16417.2		0.943	11
13	φ (PC2*LLSST_BA1980–2011_*) p (*m+t*)	31	28553.5	16415.6		0.896	8
14	φ (PC2*LLSST_BA1980–99+2005–11_*) p (*m+t*)	32	28553.2	16417.5		0.969	13
Indirect climate effects							
15	φ (*LL_CC_br _1980–2011_+ SST_CC_2yr _1980–2011_*) p (*m+t*)	32	28499.7	16386.9		0.005	9
16	φ (*LL_CC_br _1980–2011_+* [*SST_CC_2yr _1980–2011_*]^2^) p (*m+t*)	33	28487.3	16381.8		0.044	15
17	φ (*LL_CC_br _1980–2011_+ SST_CC_2yr_1980–99+2005–11_*) p (*m+t*)	33	28496.6	16387.1		0.498	15
**18**	**φ (** ***LL_CC_br _1980–2011_+ SOIyr _1980–2011_*** **) p (** ***m+t*** **)**	**32**	**28496.1**	**16384.8**		**0.002**	**9**
19	φ (*LL_CC_br _1980–2011_+* [*SOIyr _1980–2011_*]^2^) p (*m+t*)	33	28485.3	16380.6		0.058	18
20	φ (*LL_CC_br _1980–2011_+ SOIyr_1980–99+2005–11_*) p (*m+t*)	33	28486.3	16381.2		0.060	18
21	φ (*LL_CC_br _1980–2011_+ SOIw-1_1980–2011_*) p (*m+t*)	32	28525.4	16401.6		0.994	9
22	φ (*LL_CC_br _1980–2011_+* [*SOIw-1_1980–2011_*]^2^) p (*m+t*)	33	28524.1	16402.8		0.778	21
23	φ (*LL_CC_br _1980–2011_+ SOIw-1_1980–99+2005–11_*) p (*m+t*)	33	28521.1	16401.1		0.483	21
Modelling three covariates in survival							
**24**	**φ (** ***LL_CC_br + SOIyr + SST_CC_2yr _1980–2011_*** **) p (** ***m+t*** **)**	**33**	**28486.2**	**16381.1**		**0.021**	**18**
25	φ (*LL_CC_br + SOIyr +* [*SST_CC_2yr _1980–2011_*]^2^) p (*m+t*)	34	28476.8	16377.7		0.051	24
26	φ (*LL_CC_br + SOIyr + SST_CC_2yr _1980–99+2005–11_*) p (*m+t*)	34	28484.2	16382.0		0.678	24

np number of parameters estimated; DEV deviance; QAICc quasi-likelihood Akaike’s information criterion values; ΔQAIC difference between the current and the lowest QAICc model; P_ANODEV_ P-value of the ANODEV test on covariates; M_cst_ model considered as constant model when evaluating P_ANODEV_; t time; (·) constant; *m* two states of trap dependence; * interaction; + additive effect. Covariate codes are defined in Appendix S1; subindex _1980–2011_ accounts for a single period/slope while _1980–99+2005–11_ accounts for two periods/slopes; [*covariate*]^2^ denoted a covariate in a quadratic trend; in bold characters denoted the models considered.

## Discussion

The present study revealed that combined effects of climate and fishery activities in both breeding and non-breeding areas impacted negatively on the survival of a long-lived migratory seabird. Interestingly, tracking data of a hundred individuals allowed us to estimate that Cory’s shearwaters breeding at Selvagens Islands spend two thirds of the year mainly feeding in the Canary Current while breeding, and the other third migrating toward very restricted areas of the Southern Atlantic Ocean (Fig. 1). These precise spatio-temporal schedules along the entire year achieved by tracking devices became clue in selecting and delimiting specific environmental variables and potential anthropogenic threats, and ultimately allowed us to relate these precise covariates to the demography of this highly pelagic seabird.

### Modelling Demography of a Long-lived Migratory Seabird

Marine top predators, such as the Cory’s shearwater, are considered extreme K-selected species, which means that their specific life history traits, long life expectancy, delayed maturity and low reproductive rates are unavoidably linked to high adult survival [27]. Using CMR analysis we found that estimated adult survival of this mid-sized Procellariiform seabird (0.915±0.037 on average, from the model Φ. p*_m+t_*) was low compared with other Atlantic colonies (>0.93; [34], [66]), but higher than in Mediterranean populations (ranging from 0.82 to 0.90; [34], [36], [37], [67]). Survival of Cory’s shearwaters breeding at Selvagens Islands was negatively affected by a combined effect of environmental variables and fishery effort at different points of their annual life cycle. Our models reported evidence that greater longlining activity and La Niña events increased shearwater mortality in breeding and non-breeding grounds, respectively (Fig. 4). Temperature variation in the Canary Current apparently also affected negatively shearwater survival probability, although likely through an indirect effect mediated through the trophic web on which the birds depend.

Longlining has a harmful effect on the entire marine ecosystem, with significant implication for the non-targeted top predators [9]–[11]. During line setting of thousands of baited hooks, seabirds (among other marine top predators) are particularly prone to be accidentally caught while scavenging on bait, being dragged under water afterwards and finally drowned. In the Mediterranean, longlining activity is thought to be responsible for large population declines of Cory’s shearwaters at several breeding colonies [68], [69], while in the Southern Atlantic (and for extension in the entire Atlantic) Cory’s shearwater incidental bycatch was thought to occur very scarcely [50], [66], [70]–[72]. However, our results suggested that incidental mortality may have been overlooked in Atlantic waters, i.e. that longlining activity might have not only affected Mediterranean birds, but also have affected the survival of Atlantic Cory’s shearwaters (see also [51]). We suggest that the short time spent by this migratory seabird outside its breeding areas decreases the probability to be caught (as well as observed) in the Southern Atlantic Ocean, where most bycatch research has been done. Thus, Cory’s shearwater may be threatened by commercial fisheries all along its distribution, although higher impacts probably occurred at the breeding grounds [38], [73], where shearwaters spend two thirds of their annual cycle (Fig. 1).

Adult survival of Cory’s shearwaters was also negatively affected by SOI (Fig. 4b). Typically, local manifestations of the Southern Oscillation are expected to influence Cory’s shearwater wintering grounds in the Southern Atlantic, where birds spend the short non-breeding season (Fig. 1). Sustained positive values of the SOI are characterized by tropical storms and hurricanes in the Atlantic (i.e. extreme La Niña events; [23]) which have long been related to several mass mortalities and breeding failures of many marine top predators in the Southern Ocean [20], [54], [74], including seabirds in the Benguela upwelling system [75]. Indeed, our results corroborated the findings of others, which suggested that during La Niña years the greater storminess of the Southern Atlantic may cause a decrease in Cory’s shearwater survivorship [34]–[37]. Although most of these studies considered that these climate effects acted directly on winter mortality, indirect effects of SOI on wintering grounds through a trophic cascade cannot be ruled out.

In addition to longliner activity, warm sea surface temperatures around the Canary Current during the current year and the year before a given breeding period also predicted low adult survival (Fig. 4c) during the long breeding season. Although high SST has been previously found affecting negatively several seabird populations (e.g. [76]–[78]), the fact that this effect is lagged supports the idea that it is not temperature *per se*, but a mediated effect, presumably through the food web, which might trigger seabird mortality [12], [79]. Indeed, some evidence of delayed effects of SST on the trophic web of the Canary Current have been previously reported at lower trophic levels (e.g. the 6 months delay between plankton and sardine recruitment [80], [81]). Several oceanographic factors, such as positive SST values negatively influence the abundance of plankton, which in turn plays a key role in fish recruitment [82]–[84]. Thus, a reduction in productivity would decrease fish abundance, constraining the foraging opportunities of birds, and therefore increasing Cory’s shearwater mortality rate (e.g. [12], [79], [85]).

### The Selvagem’s Perspective

Cory’s shearwaters of Macaronesian Islands were exploited by indigenous inhabitants from prehistoric times (e.g. [86], [87]). More recently, regular chick harvest, with likely increasing intensity during most of the 20^th^ century, plus severe episodes of adult killings reduced dramatically the population of Selvagem Island [41]. Fortunately, after enhanced protection since the late 1970’s, the number of breeding birds built up rapidly, at *ca.* 30% per year in the early 1980’s, mainly due to the progressive recruitment of large numbers of non-breeding individuals that survived the 1975 and 1976 culls (Fig. 2; [41]). Survival estimates for that period remained relatively high and rather stable (i.e. 0.92±0.01 in 1980–1986, see Fig. 3a), which contributed to the rapid recovery of the nesting numbers. From 1987 to 1995, survival rates oscillated year-to-year, and the population growth stabilized at an average rate of 5% per year, with an estimated population of 18,100 breeding pairs in 1995 [88]. However, from that point and at least until the end of the century (i.e., 1999), survival estimates dropped to 0.86±0.02 on average, and in 1998 the numbers apparently reduced to 15,750 breeding pairs (Fig. 2; [89]), a reduction of over 13% in three years. The decrease in survival probability along this period coincides with an elevated number of hooks used by longliners in the Canary Current (Fig. 3b), which could be contributing to the significance of this covariate in our modelling. After an unknown gap of four years, Cory’s shearwater survival rates for 2005–2011 somewhat recovered (0.91±0.02) and the population apparently resumed its growth, reaching up to around 30,000 breeding pairs in 2005 (Fig. 2; [39]), and making Selvagem Grande the largest world colony of Cory’s shearwaters. Furthermore, the longliner activity in the Canary Current region seems to have decreased considerably during the last decade (Fig 3b), which may have positively contributed to the recovery of the population throughout this last period. Hence, though the Cory’s shearwater population from the Selvagens is still far from its presumed size at the beginning of the 20^th^ century, the evidence of a recovery process is very clear. However, predicted climate change and the resulting global warming could still have a negative impact both in breeding and non-breeding grounds, and continued monitoring of the dynamics of this population may be relevant to understand future trends.

### Conclusions

High adult survival and low fecundity are typical of long-lived species, and even small reductions in survival can have dramatic effects on population trends. Although survival rates reported for Cory’s shearwaters breeding at Selvagem Grande were generally higher and more stable than in most other colonies, particularly those in the Mediterranean, the amount of variation in survival explained by climatic and human-related variables was high (49.8%), considering the long period analysed. The survival of this species was moderately influenced by longlining activity and more markedly by climatic factors. Although it was encouraging that tuna longlining fishing effort declined over the last decade in the Canary Current, our results highlight the need for more research on fisheries impact on sea life in the subtropical Northeast Atlantic, where very few studies have been conducted. The results of this study also warn to the negative effects of climate at both breeding (SST) and non-breeding grounds (SOI), and it is clear that these could be aggravated in the near future, given that inter-annual variation in SST and the virulence of El Niño/La Niña events are expected to further increase [26].

From the conservation point of view, the negative impact of both longline fisheries and raised SST in the Canary Current possibly does not only concern locally breeding Cory’s shearwaters, but also wintering shearwaters from both Atlantic and Mediterranean populations that use this Current [44], [45], [90]–[92], as well as many other species inhabiting this restricted upwelling area of the subtropical Atlantic [93]–[96]. However, in terms of magnitude, the storminess generated by El Niño/La Niña phenomena in the Southern Atlantic Ocean seems to have a higher impact in the survival of Cory’s shearwaters than the effects occurring in the Canary Current. In fact, although it only represents 20% of their annual cycle, events occurring during the non-breeding period may be highly relevant for the population dynamics of Cory’s shearwaters. Overall, our results contributed to highlight the importance of considering not only breeding grounds, but also precise schedules and places visited while non-breeding for the appropriate understanding of impacts of the environment on the population dynamics of long-distance migratory species.

### Ethics Statement

All animals were handled in strict accordance with good animal practice as defined by the current European legislation. The deployment of MK7 loggers (see details above) did not take more than 10 minutes and on no occasion did it interfere with reproduction or have visible deleterious effects on study animals [97]. All work was approved by Instituto da Conservação da Natureza e da Biodiversidade and Serviço do Parque Natural da Madeira, where Selvagens Islands Nature Reserve belongs to (research permits 107/2006, 116/2007, 333/2007/CAPT).

## Supporting Information

Appendix S1 **Correlations among the explanatory covariates.** Pearson’s coefficients are placed below the diagonal and *P*-values of a *t*-test above the diagonal. Shaded areas highlight three sets of correlated covariates: a) sea surface temperatures (SST) in the breeding ground (4 consecutive periods averaged, SST_CC_2yr), b) longlining effort (LL) and SST (3 consecutive periods) in the non-breeding grounds (first and second principal components (PC) explaining respectively 47.7 and 28.8% of covariate variability), and c) Southern Oscillation Index (SOI) in two consecutive periods (current non-breeding and previous breeding periods, SOIyr). Bold type highlights the covariates retained for the analysis of survival and the correlations among them.
(DOC)
