# Supplementary material for: Combined Spatio-Temporal Impacts of Climate and Longline Fisheries on the Survival of a Trans-Equatorial Marine Migrant
Source: PLoS One. 2012 Jul 16;7(7):e40822. doi: 10.1371/journal.pone.0040822 (PMC3397926; doi:10.1371/journal.pone.0040822)
Supplement: Appendix S1 — Correlations among the explanatory covariates. Pearson’s coefficients are placed below the diagonal and P-values of a t-test above the diagonal. Shaded areas highlight three sets of correlated covariates: a) sea surface temperatures (SST) in the breeding ground (4 consecutive periods averaged, SSTCC2yr), b) longlining effort (LL) and SST (3 consecutive periods) in the non-breeding grounds (first and second principal components (PC) explaining respectively 47.7 and 28.8% of covariate variability), and c) Southern Oscillation Index (SOI) in two consecutive periods (current non-breeding and previous breeding periods, SOIyr). Bold type highlights the covariates retained for the analysis of survival and the correlations among them. (DOC) [file pone.0040822.s001.doc]

**Appendix S1.**

| Covariate | Label | **LL_CC_br** | SST_CC_br | SST_CC_w | SST_CC_br-1 | SST_CC_w-1 | **SST_CC_2y** | LL_BA_w | SST_BA_w | SST_BA_br-1 | SST_BA_w-1 | **PC1LLSST** | **PC2LLSST** | SOIw | SOIbr-1 | **SOI1yr** | **SOIw-1** |
| --- | --- | --- | --- | --- | --- | --- | --- | --- | --- | --- | --- | --- | --- | --- | --- | --- | --- |
| **Longlining effort, breeding, Apr-Sept** | **LL_CC_br** |  | 0.83 | 0.95 | 0.43 | 0.76 | **0.67** | 0.56 | 0.86 | 0.85 | 0.97 | **0.82** | **0.44** | 0.69 | 0.58 | **0.60** | **0.56** |
| SST, breeding area, Apr-Sept | SST_CC_br | 0.04 |  | 0.08 | 0.09 | 0.11 | <0.001 | 0.66 | 0.82 | 0.16 | 0.03 | 0.20 | 0.20 | 0.13 | 0.20 | 0.13 | 0.20 |
| SST, breeding area, previous Dec-previous Feb | SST_CC_w | 0.01 | 0.30 |  | 0.01 | 0.37 | <0.001 | 0.64 | 0.86 | 0.53 | 0.64 | 0.93 | 0.45 | 0.57 | 0.60 | 0.55 | 0.71 |
| SST, breeding area, previous Apr-previous Sept | SST_CC_br-1 | 0.14 | 0.29 | 0.42 |  | 0.07 | <0.001 | 0.42 | 0.25 | 0.85 | 0.65 | 0.38 | 0.91 | 0.45 | 0.58 | 0.48 | 0.19 |
| SST, breeding area, 2^nd^ previous Dec-2^nd^ -previous Feb | SST_CC_w-1 | -0.06 | 0.28 | 0.16 | 0.32 |  | <0.001 | 0.83 | 0.34 | 0.50 | 0.96 | 0.61 | 0.67 | 0.07 | 0.07 | 0.09 | 0.86 |
| **Integrative SST index, breeding area, 2 years** | **SST_CC_2y** | **0.08** | 0.74 | 0.63 | 0.79 | 0.56 |  | 0.44 | 0.35 | 0.47 | 0.18 | **0.24** | **0.65** | 0.10 | 0.16 | **0.10** | **0.21** |
| Longlining effort, wintering area, previous Dec-previous Feb | LL_BA_w | -0.11 | 0.08 | 0.09 | 0.15 | 0.04 | 0.14 |  | 0.19 | 0.08 | 0.01 | <0.001 | <0.001 | 0.14 | 0.63 | 0.63 | 0.37 |
| SST, wintering area, previous Dec-previous Feb | SST_BA_w | 0.03 | 0.04 | 0.03 | 0.21 | 0.17 | 0.16 | 0.24 |  | 0.21 | 0.29 | 0.02 | 0.23 | 0.57 | 0.52 | 0.52 | 0.76 |
| SST, wintering area, previous Apr-previous Sept | SST_BA_br-1 | 0.04 | 0.25 | -0.11 | 0.03 | 0.12 | 0.13 | 0.31 | 0.22 |  | <0.001 | <0.001 | <0.001 | 0.03 | 0.59 | 0.16 | 0.51 |
| SST, wintering area, 2^nd^ previous Dec-2^nd^ previous Feb | SST_BA_w-1 | 0.01 | 0.38 | 0.08 | 0.08 | 0.01 | 0.23 | 0.47 | -0.19 | 0.54 |  | <0.001 | 0.29 | 0.05 | 0.33 | 0.07 | 0.29 |
| **Integrative index (PC1: LL+SST), wintering area** | **PC1LLSST** | **-0.04** | 0.23 | 0.02 | 0.16 | 0.09 | **0.21** | 0.86 | 0.41 | 0.71 | 0.66 |  | **1.00** | 0.03 | 0.90 | **0.30** | **0.88** |
| **Integrative index (PC2: LL+SST), wintering area** | **PC2LLSST** | **-0.14** | -0.23 | 0.14 | 0.02 | -0.08 | **-0.08** | 0.51 | -0.22 | -0.58 | -0.19 | **0.00** |  | 0.47 | 0.46 | **0.43** | **0.13** |
| SOI, previous Dec-previous Feb | SOIw | -0.07 | 0.26 | 0.10 | 0.13 | 0.31 | 0.28 | 0.26 | -0.10 | 0.37 | 0.53 | 0.39 | -0.13 |  | <0.001 | <0.001 | 0.24 |
| SOI, previous Apr-previous Sept | SOIbr-1 | -0.10 | 0.23 | 0.09 | 0.10 | 0.31 | 0.25 | -0.09 | -0.11 | 0.10 | 0.17 | -0.02 | -0.13 | 0.71 |  | <0.001 | 0.93 |
| **Integrative SOI index, 1 year** | **SOI1yr** | **-0.10** | 0.26 | 0.11 | 0.13 | 0.34 | **0.29** | 0.09 | -0.12 | 0.25 | 0.37 | **0.19** | **-0.15** | 0.92 | 0.93 |  | **0.58** |
| **SOI, 2^nd^previous Dec-2^nd^previous Feb** | **SOIw-1** | **-0.11** | 0.22 | 0.07 | 0.23 | -0.03 | **0.22** | 0.16 | 0.06 | -0.12 | -0.19 | **0.03** | **0.27** | -0.21 | 0.02 | **-0.10** |  |

* Breeding area includes the Canary Current (20º-35ºN, 10º-20ºW), while the non-breeding area includes both the Benguela and the Agulhas Currents (15º-40ºS, 5º-25ºE and 15º-40ºS, 25º-45ºE, respectively).
